# Prognostic role of hERG1 Potassium Channels in Neuroendocrine Tumours of the Ileum and Pancreas

**DOI:** 10.3390/ijms231810623

**Published:** 2022-09-13

**Authors:** Jessica Iorio, Lorenzo Antonuzzo, Emanuela Scarpi, Massimo D’Amico, Claudia Duranti, Luca Messerini, Clotilde Sparano, Damiano Caputo, Daniele Lavacchi, Domenico Borzomati, Alice Antonelli, Lorenzo Nibid, Giuseppe Perrone, Alessandro Coppola, Roberto Coppola, Francesco di Costanzo, Elena Lastraioli, Annarosa Arcangeli

**Affiliations:** 1 Department of Experimental and Clinical Medicine, University of Florence, 50134 Florence, Italy; 2 Medical Oncology, Azienda Ospedaliero-Universitaria Careggi, 50134 Florence, Italy; 3 Unit of Biostatistics and Clinical Trials, IRCCS Istituto Romagnolo per lo Studio dei tumori (IRST) “Dino Amadori”, 47014 Meldola, Italy; 4 DI.V.A.L Toscana Srl, 50019 Sesto Fiorentino, Italy; 5 Endocrinology Unit, Department of Experimental and Clinical Biomedical Sciences “Mario Serio”, University of Florence, 50134 Florence, Italy; 6 General Surgery, Campus Bio-Medico University, 00128 Rome, Italy; 7 Fondazione Policlinico Universitario Campus Bio-Medico, 00128 Rome, Italy; 8 Pathology Unit, Campus Bio-Medico University, 00128 Rome, Italy; 9 Complex Dynamics Study Centre (CSDC), University of Florence, 50100 Florence, Italy

**Keywords:** hERG1 channel, neuroendocrine tumours, ileum, pancreas, prognosis

## Abstract

hERG1 potassium channels are widely expressed in human cancers of different origins, where they affect several key aspects of cellular behaviour. The present study was designed to evaluate the expression and clinical relevance of hERG1 protein in cancer tissues from patients suffering from neuroendocrine tumours (NETs) of ileal (iNETs) and pancreatic (pNETs) origin, with available clinicopathological history and follow-up. The study was carried out by immunohistochemistry with an anti-hERG1 monoclonal antibody. In a subset of samples, a different antibody directed against the hERG1/β1 integrin complex was also used. The analysis showed for the first time that hERG1 is expressed in human NETs originating from either the ileum or the pancreas. hERG1 turned out to have a prognostic value in NETs, showing (i) a statistically significant positive impact on OS of patients affected by ileal NETs, regardless the TNM stage; (ii) a statistically significant positive impact on OS of patients affected by aggressive (TNM stage IV) disease, either ileal or pancreatic; (iii) a trend to a negative impact on OS of patients affected by less aggressive (TNM stage I-III) disease, either ileal or pancreatic. Moreover, in order to evaluate whether ERG1 was functionally expressed in a cellular model of pNET, the INS1E rat insulinoma cell line was used, and it emerged that blocking ERG1 with a specific inhibitor of the channel (E4031) turned out in a significant reduction in cell proliferation.

## 1. Introduction

Neuroendocrine tumours (NETs) are a heterogeneous family of rare neoplasms originating from specialised neuroendocrine cells throughout the body. NETs represent one of the most frequent malignancies of the small bowel, in particular the ileum, although they can also develop in pancreas, stomach, lung, thymus and colon among others. NETs arising in the pancreas and in the ileum share several pathological and biological features but, since they also have important differences in both pathogenesis and treatment [1,2] they are generally considered separately [3]. The embryologic origin of the diffuse neuroendocrine system is still debated [4], and while originally it was thought that all of these cells derived from neural crests, recently it was shown that neuroendocrine intestinal cells share the same endodermal origin as absorbing, goblet and Paneth cells [5]. 

NETs may present with a wide range of morphological, functional and behavioural characteristics. Moreover, clinical signs are not specific and due to their low frequency; initially NETs are not considered for differential diagnosis. For these reasons, the management of NETs is quite challenging. Nowadays, the clinical management of NETs is mainly based on the WHO classification, where NETs are divided according to the proliferation index (Ki67 expression and mitotic index) [6]. Indeed, accurate biomarkers to guide clinical decisions are currently lacking, which represents a critical limitation for NETs’ prognostic and predictive evaluations. Neuroendocrine markers [7], either specific (i.e., substances produced by functioning NETs only) or non-specific (i.e., substances produced by all NETs) [8,9,10] have been proposed as biomarkers, but they are not yet validated. Hence, the identification of sensitive and specific diagnostic and prognostic biomarker useful for NETs’ clinical management is still an unmet need.

In recent decades, mounting evidence has pointed out at ion channels as novel biomarkers in human cancers [11]. Among them, potassium channels exert a key role [12]. In particular, the human ether-á-go-go–related gene (hERG1) is expressed in different types of human solid cancers, while absent in healthy counterparts [13,14,15,16,17,18,19,20,21]. Physiologically, hERG1 is expressed in the heart, where it regulates the cardiac action potential [22], in different neuronal populations, where it modulates excitability [23], as well as in muscle cells, where it plays a role in regulating contractility [24]. Interestingly, hERG1 is also expressed in endocrine cells, where it acts as a regulator of hormone secretion through the modulation of action potential frequency [25,26]. In tumours, the presence of hERG1 channels contribute (i) to clamping the resting potential at rather less negative values compared to normal cells, which represents a fundamental prerogative for cells destined to unlimited growth [27], and (ii) to trigger intracellular signalling pathways involved in cell survival, proliferation, motility and invasion [28]. This occurs through the formation of a molecular complex with the β1 subunit of integrin receptors [29]. Overall, hERG1 could represent a novel cancer biomarker in different tumours, including those of the gastrointestinal tract [21,30,31,32].

Based on these premises the aim of the present work was to evaluate the expression and clinical relevance of hERG1 potassium channel in ileal and pancreatic neuroendocrine tumours (iNETs, pNETS), which we here considered separately since they are commonly regarded as different pathologies [1,2,3]. 

## 2. Results

**Figure 1:**
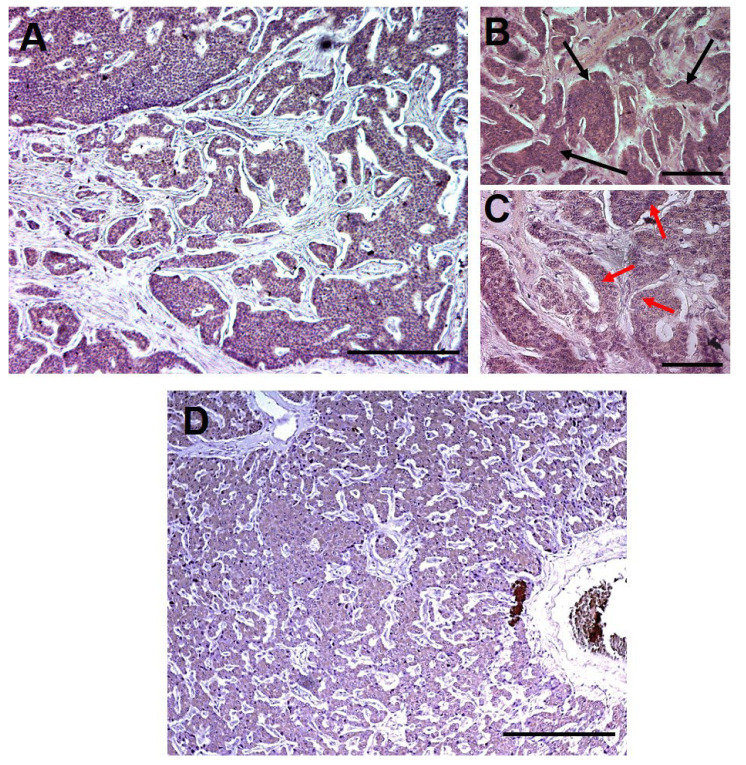
**Immunohistochemistry for hERG1 in NET specimens.** Immunohistochemical detection of the hERG1 protein in representative specimens of NET using anti-hERG1 monoclonal antibody. (**A**) Ileal NET; (**B**). Higher magnification of the representative sample shown in (**A**). Large groups of tumour cells highly expressing the hERG1 channel (as witnessed by the brown colour) are indicated by black arrows. (**C**) High power microphotograph of the representative sample reported in (**A**,**B**). hERG1 positive cells are identified by the brown precipitate located in the cytoplasm while nuclei (red arrows) are negative, counterstained in blue by haematoxylin. (**D**) Pancreatic NET. Scale bar: 200 µm, Magnification 10× (**A**,**D**), 20× (**B**) and 40× (**C**).

The expression of hERG1 potassium channel was evaluated by immunohistochemistry in a cohort of 31 iNET and 59 pNET with the mAb hERG1 antibody (Figure 1), as described in Materials and Methods. Clinico-pathological characteristics, including age, sex, disease stage and treatments were retrieved from the patients’ medical records and referring physicians and are reported in Table 1 and Table 2 for iNET and pNET, respectively.

### 2.1. hERG1 Channel Expression in Ileal NETs

A detailed evaluation of the specimens was carried out for each sample estimating the percentage of hERG1 expressing tumour cells per microscopic field. A positive iNET sample is shown in Figure 1A: as it can be observed, hERG1 channel expression (indicated by the brown colour) is present throughout the tumour while stroma turned out to be negative. Overall, 26 patients out of 31 (83.9%) were positive for hERG1 expression while 5 (16.1%) were negative. 

#### 2.1.1. Association of hERG1 Expression and Clinical Features in Ileal NETs

In order to evaluate eventual association between hERG1 expression and clinical-pathological features, a statistical analysis was performed taking into account positivity/negativity as well as the median value of positive cells (Table S1). As reported in the table, no statistically significant association emerged. 

#### 2.1.2. Survival Analyses in Ileal NETs

Median follow-up was 92 months (range 1–336). Overall, 12 patients died during the period of the investigation (6 patients died for disease progression while the remaining 6 died for unrelated causes, see also Figure S1). In univariate analyses among routinely evaluated parameters, Ki67 turned out to be statistically associated with Overall Survival (OS) (*p* = 0.036, Table 3). More interestingly, using a cut-off of 40% (calculated through Receiver operating characteristic (ROC) analysis as detailed in Materials and Methods) hERG1 expression turned out to be significantly associated with OS (*p* = 0.020, HR: 0.23, 95% CI: 0.07–0.80) with a positive impact (Table 3). Interestingly, hERG1 behaved as a protective factor in iNETs. 

**Figure 2:**
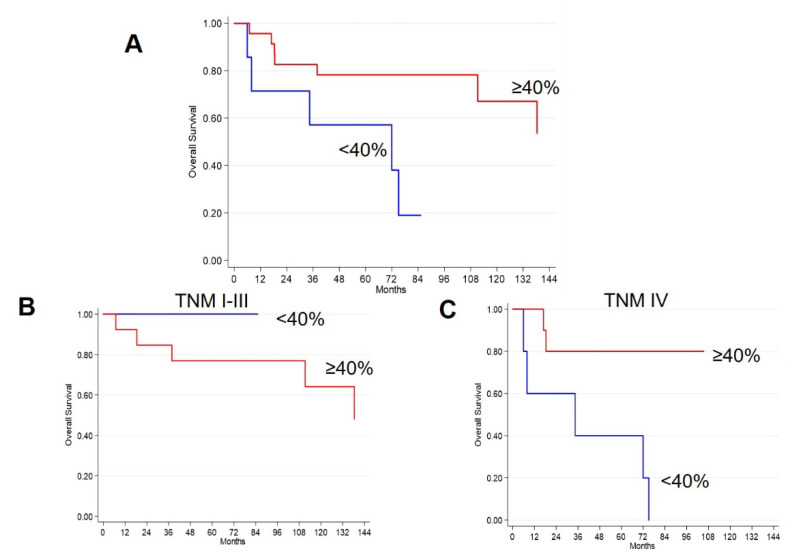
Kaplan-Meier plots of OS in iNET patients according to hERG1 expression (cut-off: 40%). (**A**) Whole cohort. (**B**) TNM I-III; (**C**) TNM IV. Blue curves: hERG1 negative samples (<40%), Red curves: hERG1 positive samples (≥40%).

In Figure 2, Kaplan Meier plots are reported; they refer to the whole cohort (panel A) as well as subdividing the patients according to TNM (panels B and C), with a 40% cut-off. It is evident that patients with high hERG1 expression (≥40%, red curves) have a longer OS with respect to those with lower expression of the channel (blue curves) in the whole cohort and in the TNM IV group. In the TNM I-III cohort the trend is opposite, but it must be pointed out that in this case the number of events is low (n = 5) and none of the hERG1 negative patients (<40%) died.

Table S2 shows the analysis performed in TNM IV patients, in which the *p* value is more statistically significant than in the whole cohort and ki67 failed to associate with OS. 

In univariate analyses, no association with progression free survival (PFS) emerged (Tables S3 and S4, Figure S2). 

### 2.2. hERG1 Channel Expression in Pancreatic NETs

As performed for iNET, the percentage of hERG1 expressing tumour cells per microscopic field was evaluated in all pNET samples. A representative positive sample is shown in Figure 1B: hERG1 is expressed in the tumour while stromal tissue is negative. Overall, 37 patients out of 59 (62.7%) were positive for hERG1 using a cut-off equal to 0% (i.e., positive vs negative); applying the same cut-off as for iNET (40%, calculated through ROC analysis as detailed in Materials and Methods) 18 samples (30.5%) were classified as positive. 

#### 2.2.1. Association of hERG1 Expression and Clinical Features in Pancreatic NETs

The same approach used for iNETs was applied to the pNET cohort to evaluate possible association between hERG1 expression and clinical-pathological features (Table 4). Interestingly, hERG1 expression was associated with TNM stage, with higher levels of the channel in TNM II patients (*p* = 0.011). 

#### 2.2.2. Survival Analyses in Pancreatic NETs

Median follow-up was 103 months (range 2–218). Overall, 10 patients died during the period of the investigation (6 patients died for disease progression while the remaining 4 died for causes unrelated to pNET). In univariate analyses among routinely evaluated parameters, the presence of metastases at diagnosis, radical surgery and Ki67 turned out to be statistically associated with OS (*p* = 0.002, *p* = 0.002, *p* < 0.0001, respectively) (Table 5). 

**Figure 3:**
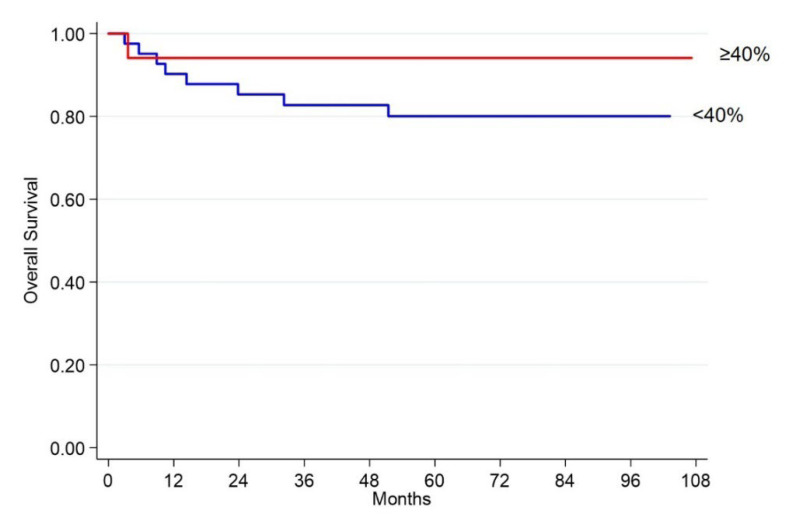
Kaplan-Meier plots of OS in pNET patients according to hERG1 expression (cut-off: 40%). Blue curves: hERG1 negative samples (<40%), Red curves: hERG1 positive samples (≥40%).

Although significance was not reached, the Kaplan Meier plot shows that also in this case, hERG1 positive patients have a longer OS (*p* = 0.169, Table 5 and Figure 3). As performed in iNETs, analyses were carried out using a 40% cut-off (calculated through ROC analysis) as well as discriminating between positive and negative samples.

In univariate analyses, no association with PFS emerged in TNM I-III patients (Table S5) while in TNM IV patients only Ki67 turned out to be significantly associated with PFS (Table S6). From the Kaplan-Meier plots, it can be observed that TNM I-III hERG1 positive patients (≥40%) have a longer PFS, although significance is not reached (Figure S3). In TNM IV patients, an opposite trend is observed but it must be pointed out that the number of events in the hERG1-positive group is quite low (n = 2).

### 2.3. Survival Analyses in the Whole Cohort

Finally, the eventual impact of hERG1 expression on OS was evaluated in the whole cohort, taking together iNETs and pNETs. The results of the analysis are reported in the following table (Table 6). 

In TNM IV NETs hERG1 is confirmed to act as a protective factor since patients with high hERG1 expression (≥40) have a longer OS, while in TNM I-III patients, statistical significance is not reached. More strikingly, in these patients, hERG1 represents a negative prognostic factor since HR is greater than 1. 

### 2.4. hERG1 Is Functionally Expressed in Insulinoma (INS1E) Cells and hERG1 Blockers Impair cell Proliferation In Vitro

We then evaluated whether ERG1 was functionally expressed in a cellular model of well-differentiated pNETs. To this purpose, we used the rat insulinoma cell line INS1E, which is characterised by features of normal pancreatic beta cell (i.e., a high insulin content and responsiveness to glucose within the physiological range). 

**Figure 4:**
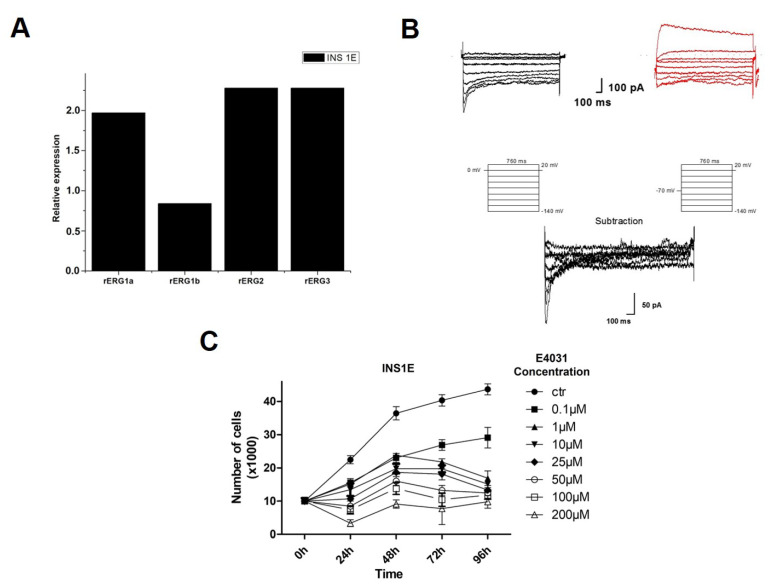
(**A**) Graph representing rERG1a, rERG1b, rERG2 and rERG3 mRNA expression in INS1E cells is reported; Relative expression is reported in folds value. (**B**) Representative rERG current registered in INS1E cells. Whole-cell inward rectifying current traces (upper traces) elicited by the stimulation protocols shown in the middle of the figure. The extracellular solution contained 40 mm K^+^, giving an E_k_ around −30 mV. Cell was conditioned at 0 mV, before applying each test pulse (traces and protocol on the left) and subsequently was conditioned at −70 mV, before applying each test pulse (red traces and protocol on the right). At the holding potential of −70 mV all ERG channels are completely closed, and the application of the test pulses is not able to evoke the quote of the inward current carried by ERG channels. By subtracting the currents in panels (**A**,**B**), we obtained the pure inward component of I_RERG_ (lower traces). (**C**) ERG Effects of E4031 on proliferation of INS1E cells. Data are reported as mean ± SEM of three independent experiments.

As a preliminary step, RQ-PCR (real-time reverse transcription–polymerase chain reaction) experiments showed that *rERG1a, rERG1b, rERG2 and rERG3* are all expressed in INS1E cells (Figure 4A).

The expression of ERG in INS1E cells was then confirmed by patch clam technique (Figure 4B). Using the protocol with a holding potential of 0 mV, we recorded the traces shown on the left. In this way, we were able to observe the appearance of an inward rectifying current. This current disappears partially when we use the protocol with a holding potential of −70 mV (red traces on the right). Indeed, with this potential, all ERG channels are completely closed and the application of the test pulses is not able to evoke the quote of the inward current carried by ERG channels. The I_ERG_ component can be better appreciated after the software subtraction of the currents recorded at the two different holdings (lower traces). Overall, this component, with a mean current density equal to 21.6 ± 9.7 pA/pF was registered in 9 cells. 

Finally, the effect of the ERG1 blocker E4031 on cell viability was assessed (Figure 4C): the treatment caused a time- and dose-dependent inhibition of cell proliferation, which was statistically significant at all time points (Table S7).

**Figure 5:**
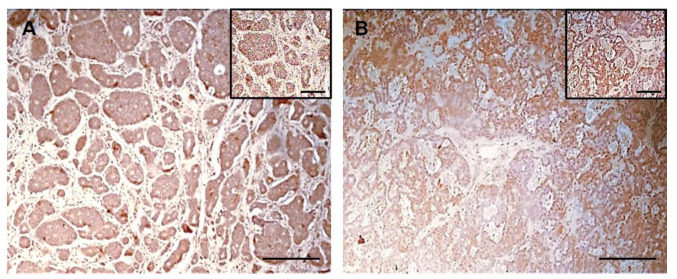
**Immunohistochemistry for hERG1/β1 Integrin complex in NET samples.** Immunohistochemical detection of the hERG1/β1 Integrin complex in representative tissues sample of NET using scDb hERG1-β1 antibody. (**A**) Ileal NET; (**B**) Pancreatic NET. Scale bar: 200 μm.

The effects of E4031 on cell viability of the insulinoma cell line suggest that hERG1 could be exploited as a therapeutic target also in NETs, at least in early stages pNETS, which are well modelled by the INS1E cells and where hERG1 is highly expressed (see Table 5). However, hERG1 blockers cannot be proposed for therapeutic purposes due to the challenges deriving from hERG1 physiologic expression in the heart [29,33]. Hence, we assessed the expression of the tumour specific complex formed by hERG1 and β1 integrin subunit, which could be safely targeted by a single chain diabody directed against the hERG1/β1 complex (scDb-hERG1/β1) [33]. To this purpose, the same diabody (scDb-hERG1/β1) was used to perform immunohistochemistry experiments on two cohorts of 10 iNETs and 10 pNETs, respectively. Representative images, showing a positive staining for hERG1/β1 complex, are reported in Figure 5. 

Overall, the hERG1/β1 complex was detected in 4 out of 10 iNET samples using a 40% cut-off and in 6 out of 10 iNETs using a 1% cut-off. In pNET samples 6 out of 1 were classified as positive with both cut-offs. A statistically significant correlation between hERG1 expression and hERG1/β1 complex for both iNETs and pNETs emerged (*p* = 0.0012, R^2^ = 0.7519 and *p* = 0.0008, R^2^ = 0.8772, respectively). 

## 3. Discussion

Due to the lack of proper biomarkers to identify and characterise NETs and of potentially effective treatments, despite huge efforts achieved in recent years, the management of NETs is still challenging [34]. In the present paper we aimed at overcoming this issue, analysing the expression and clinical relevance of the hERG1 potassium channel in two clinical cohorts of iNETs and pNETs, respectively.

We showed for the first time that: (1) the hERG1 potassium channel is expressed in human neuroendocrine tumours originating from either the ileum (iNETs) or the pancreas (pNETs); (2) in iNETs, hERG1 expression showed a statistically significant positive impact on OS in TNM stage I-IV cases; (3) in pNETs hERG1 expression was significantly higher in early TNM stages (stage II), and showed a positive impact on OS in TNM stage I-IV cases, although not reaching the statistical significance; (4) in the whole cohort (iNETs plus pNETs) hERG1 expression showed a statistically significant positive impact (HR < 1) on OS of patients affected by an aggressive (TNM stage IV) disease, and a negative impact (HR > 1) on OS of patients affected by a less aggressive (TNM stages I-III) disease, which did not reach the statistical significance; (5) in both iNETs and pNETs, hERG1 is present as a complex with the β1 integrin subunit, and 6) blocking hERG1 in an insulinoma cell line significantly reduces cell proliferation. 

As stated in the Introduction, the identification of specific tissue tumour markers still represents a huge medical need, since neither non-specific NET biomarkers (e.g., chromogranin-A (CHGA), Neuron Specific Enolase (NSE), Pancreatic Polipeptide (PP), Human Chorionic Gonadotropin (HCG), and Alpha Fetoprotein (AFP) [35]), or specific circulating NET biomarkers (e.g., gastrin, insulin, glucagone, somatostatin and vasoactive intestinal peptide (VIP)) have shown a validated clinical relevance [35]. 

To fill this gap, we have studied a novel class of cancer biomarkers, i.e., ion channel proteins. The expression and role of calcium channels in NETs have been elucidated (see [36] for a comprehensive review), and the large-conductance Ca^2+^-activated K^+^ channel (BK_Ca_) is expressed in the human somatostatinoma QGP-1 cell line and that the regulatory γ1 subunit promotes cell proliferation [37]. However, to the best of our knowledge, no data have been published about hERG1 expression and clinical significance in human neuroendocrine tumours. In the last decades, it has been demonstrated that hERG1 potassium channels are overexpressed in several human solid tumours [14,16,17,18,20,21,38,39] and numerous reports also showed their clinical relevance [20,21,30,31,40,41,42,43]. hERG1 expression is frequently associated with poor prognosis but the contrary is also true. In particular in breast tumours where hERG1 channel expression contributes to identify patients with better outcome [17] and the same happens in patient bearing metastatic colorectal cancer and treated with bevacizumab [31] differently from what is observed in non-metastatic patients [44]. In this paper, applying a 40% cut-off (calculated through ROC analysis), hERG1 expression was found to be significantly associated with longer overall survival in both iNETs and pNETs, a finding which differs from that which is reported for other tumours of the gastrointestinal (GI) tract [21,44]. This apparent contradiction can be reconciled (i) considering the different origin of NETs compared to GI carcinomas (neuroendocrine vs epithelial) as well as (ii) the physiological role exerted by hERG1 on firing and in normal pancreatic beta cells [45,46]. hERG1 channels are also involved in mouse development, especially in the central nervous system, retina and skeletal muscle, among other tissues [47,48]. 

The connection between hERG1 and neuronal lineage in adults and during development was established long ago [28,49]; in addition, it was demonstrated that hERG1 is highly expressed in neuroblastoma cells across several species from mouse to man [50,51]. The physiological role of hERG1 in neuroendocrine healthy cells of the pancreas strongly suggests that the expression of hERG1 in NETs could represent a feature of well differentiated cells thus accounting for a better outcome of the patients. Consistently, hERG1 expression is higher in early stages pNETs, and the maintenance of the channel in advanced, metastatic NETs would give to these patients an increased chance of survival. Alongside speculations, our data showing that hERG1 expression is a positive prognostic factor of OS in NET patients, suggest that the evaluation of hERG1 expression could be applied in the clinical setting to identify patients at higher risk (i.e., those with low hERG1 expression).

In this paper we also performed experiments with an insulinoma cell line, and we showed that ERG K^+^ channels are expressed in this model and that the treatment with a specific inhibitor (E4031) caused a sharp reduction in cell viability. Although still preliminary and suffering from the limitations intrinsically present in cellular models (no stromal components and cell interactions), these data raise the possibility of introducing hERG1 inhibitors in the clinical settings after proper in vitro and in vivo validation. However, the possibility of directly targeting the channel as a therapeutic strategy cannot be pursued due to the severe cardiotoxic side effects that hERG1 blockade may cause [20]. In order to overcome these problems, our group has recently unrevealed a novel tumour specific target, represented by the hERG1/β1 integrin complex and developed a tool, in the format of a single chain diabody (scDb-hERG1/β1), able to target this macromolecular complex [33]. The specificity of scDb assessed through a Peptide ELISA [33] allow us to discriminate between hERG1 channel and hERG1/β1 integrin complex. For these reasons, we also provide here preliminary evidence that the hERG1/β1 integrin complex is expressed in both pNETs and iNETs. These results suggest that hERG1 positive expression in NETs relies on the expression of the complex, more than of the channel *per se*, which should be validated in a larger cohort. 

Data presented here are the results of a pilot study. The main limitations of our study are represented by: (1) the retrospective study design; (2) the reduced sample size; (3) the lack of selection and uniformity of patients, especially in terms of the different treatments administered within iNETs and pNETs. Nevertheless, our data suggest a positive prognostic role for hERG1 K+ channel that might be sustained by its structural conformation complexed with β1 integrin, which we have confirmed for the first time in NETs, using a new recombinant antibody targeting the hERG1/β1 complex. Overall, these findings identify a useful new tissue biomarker for iNETs and pNETs, which could be exploited for prognostic as well as therapeutic purposes in the future. 

## 4. Materials and Methods

**Study Design.** A retrospective monocentric study was performed on a cohort of 31 patients suffering from ileal NET (iNET) diagnosed between 1993 and 2015 on surgical specimens and treated at Azienda Ospedaliero-Universitaria Careggi (Florence). 

For pancreatic NET (pNET), a retrospective multicentric study was performed on a cohort of 59 patients diagnosed with pNET between 1999 and 2015 and treated at Campus Biomedico University of Rome.

**Patients and tissue specimens**. Tissue samples were obtained from the Department of Experimental and Clinical Medicine, University of Florence and from the Pathology Division, Campus Biomedico University of Rome. Diagnosis and histological grading were assessed in all cases using standard criteria by experienced pathologists (LM and GP).

All cases had sufficient material for the pathological and molecular analysis and were thus eligible for the study. At the time of diagnosis, all of the patients included in the study were older than 18 years and provided consent. A total of 31 iNET patients (15 males, 16 females) with a median age of 63 years (range: 40–86 years) were analysed. For pNET, a cohort of 59 patients (30 males, 29 females) with a median age at diagnosis of 63 years (range: 25–79 years) was analysed.

**Immunohistochemistry.** Immunohistochemistry was performed as previously reported by our group [44], using an anti-hERG1 monoclonal antibody (MCK Therapeutics; Florence, Italy; patent number IT1367861) at 1:200 dilution and scDb- hERG1/β1 antibody (MCK Therapeutics; Florence, Italy; patent number IT102017000083637, granted for Italy on 9 October 2019; internationally extended in USA, Europe, Canada, China, United Emirates, Australia, Japan and South Korea) 20 µg/mL as in [33]. Briefly, after dewaxing and re-hydrating the sections, slides were incubated overnight at 4 °C with the above-mentioned primary antibody. The following day, immunostaining was performed with PicTure Max kit and DAB (Invitrogen; Carlsbad, CA, USA). Samples were then analysed using Leica DMR light microscope (Leica; Wetzlar, Germany) by two independent operators (EL and JI); for each sample, the percentage of stained tumour cells was evaluated. The specificity of the antibodies was already demonstrated in previous work and it was shown that hERG1 antibody selectively recognises the hERG1A isoform [39]. The specificity of scDb was assessed through a Peptide ELISA and has been published elsewhere [33,52].

**Statistical analysis.** Data were summarized using descriptive statistics (absolute and relative frequency for categorical variable whereas median and interquartile range for continuous variable).

Overall Survival (OS) was calculated from the date of diagnosis to the date of death or the date of last follow-up. Progression-free survival (PFS) was calculated as the time between the date of start of treatment and the first date of progression or death, whichever comes first, or last tumour evaluation.

Receiver operating characteristic (ROC) curve analysis was used to determine the best threshold of expression of hERG1 potassium channel. 

Comparisons of the continuous variables and the clinical-pathological characteristics were carried out using the median test.

OS and PFS were evaluated using Kaplan-Meier method and 95% confidence interval (95% CI) were estimated using Greenwood formula. Logrank test was used to compare survival curves. Hazard ratio and relative 95% CI were estimated using univariate Cox regression.

Median follow-up was estimated using the reverse Kaplan-Meier estimator.

Correlation with Pearson Coefficient was performed to evaluate the linkage between the expression of hERG1 and hERG1/β1 complex (significant *p* value < 0.05).

All tests were two sided, and *p* < 0.05 was considered significant. Statistical analysis was conducted using SAS Statistical Software release 9.4 (SAS Institute; Cary, NC, USA).

**Cell cultures.** INS1E cells were cultured in Roswell Park Memorial Institute (RPMI) 1640 Medium (Sigma-Aldrich; St. Louis, MO, USA) with sodium bicarbonate, supplemented with 10% foetal bovine serum, 2 mmol/L L-glutamine, 1 mM sodium pyruvate, 10 mM HEPES, 50 mM 2-mercaptoethanol, 100 U/mL penicillin and 100 µg/mL streptomycin. Cells were maintained at 37 °C and 5% CO_2_. 

**Total RNA extraction and Real Time PCR.** Total RNA was extracted following the TRIzol^®^ Reagent (ThermoFisher; Waltham, MA, USA) protocol. *rERG1a, rEERG1b, rERG2* and *rERG3* mRNAs were quantified by real-time quantitative polymerase chain reaction (RQ-PCR), using the PRISM 7700 sequence detection system (Applied Biosystems; Carlsbad, CA, USA) and the SYBR Green PCR Master Mix Kit (Applied Biosystems; Carlsbad, CA, USA) as in [53,54]. 

Primers used are the following:

rERG1a-F: 5′-TGGAGAAGGA CATGGTAGGG-3′
rERG1a-R: 5′-GTCAGGTCCA CATCCACCAC-3′
rERG1b-F: 5′-GGAAGGAGAG CAGGACAGG-3′
rERG1b-R: 5′-GATGGTCCAG CGGTGTATTC-3′
rERG2-F: 5′-AGATTGGAGT CCCGTGTGTC-3′
rERG2-R: 5′-TCCCACCAGAA GCGTAGACT-3′
rERG3-F: 5′-CGTCTTCCTTT ATCTCCTCC-3′
rERG3-R: 5′-CTGTAAGATGG CCTGGATGT-3′
GAPDH-F: 5′-AGACAGCCGCATCTTCTTGT-3′
GAPDH-R: 5′-CTTGCCGTGGGTAGAGTCAT-3′

The relative expression of *rERG1A*, *rERG1B*, *rERG2* and *rERG3* was calculated by using comparative threshold cycle method. *GAPDH* housekeeping gene was used as standard reference. Standard curves were determined preparing serial dilution of cDNA from whole rat brain serving as positive control. Amplification of rat liver was performed as a further negative control. 

**Electrophysiology.** Cells, seeded on 35-mm Petri dishes (Corning Inc; Corning, NY, USA), were patched at room temperature after 2 days of culture, and traces were recorded with the patch-clamp amplifier MultiClamp 700A (Axon Instruments; Foster City, CA, USA) using the whole-cell configuration. Measurements of the currents were performed in current voltage clamp. The pipettes used (borosilicate glass, Harvard Apparatus, Kent, UK) had resistances ranging between 3 and 5 MΩ. Gigaseal resistances were in the range 1 to 10 GΩ. Whole-cell currents were filtered at 1 to 3 KHz. For data acquisition and analysis, the pClamp 8 and Axoscope software (Axon Instruments; Foster City, CA, USA) and Origin (OriginLab; Northampton, MA, USA) were routinely used. Extracellular solutions were delivered with hypodermic needles inserted into a capillary with a small hole (inner diameter, 0.4 mm), positioned near the cell under study. The extracellular solution with high potassium (high K_o_ solution [K]_0_ = 40 mM) contained (in mM) NaCl 95, KCl 40, CaCl_2_ 2, MgCl_2_ 2, HEPES-NaOH 10, and glucose 5, pH 7.4. The standard pipette solution at [Ca^2+^] = 10^−7^ M contained (in mM) K^+^ Aspartate 130, NaCl 10, MgCl_2_ 2, CaCl_2_ 2, EGTA-KOH 10, and HEPES-KOH 10, pH 7.4. 

For the measurement of inward rectifying currents, a protocol consisted of 9 episodes, each with one preconditioning step at 0 mV, followed by steps ranging from +20 mV to −140 mV (with 20 mV intervals) was used. A similar protocol, but with a preconditioning step at −70 mV, was then applied. This voltage potential keeps the ERG channels in the closed conformational state and the following steps of the protocol allow us to induce the remaining inward currents but are not enable to elicit the ERG current. The current resulting from the software subtraction of the currents obtained by the two applied protocols is therefore identified as rERG1.

**Cell viability assay.** Cell viability was assessed through the Trypan Blue exclusion test (Sigma-Aldrich; St. Louis, MO, USA): cells were seeded at 1 × 10^4^/well in 96 well plates (Corning Inc; Corning, NY, USA) in complete medium and incubated for 24 h before E4031 (ERG specific inhibitor) addition. Cells were further incubated for different times (24 h, 48 h, 72 h and 96 h) and with different E4031 concentration (range 0–200 µM). When the Trypan Blue exclusion test was applied, cells were harvested and counted using a Bürker chamber. All experiments were performed in triplicate.

## Figures and Tables

**Table 1:** Demographic and clinical features of iNET ^1^ patients.

	N. (%)
**Age**, years: median value (range, IQR)	63 (40–86, 54–74)
<70	22 (71.0)
≥70	9 (29.0)
**Gender**	
Female	16 (51.6)
Male	15 (48.4)
**TNM at diagnosis**	
I	0 (0.0)
II	5 (16.1)
III	11 (35.5)
IV	15 (48.4)
**Metastases at diagnosis**	
No	16 (51.6)
Yes	15 (48.4)
**Radical surgery**	
No	13 (41.9)
Yes	18 (48.1)
**Metastatic sites**	
0	16 (51.7)
1	25 (80.6)
>1	6 (19.4)
**Ki67 (%)**	
<3	15 (48.4)
3–20	14 (45.2)
>20	2 (6.4)
**Relapse**	
No	20 (64.5)
Yes	11 (35.5)
Liver	4
Lung	1
Limphnodes	2
Peritoneum	2
Other	1
Unknown/missing	1
**SSA ^2^ Receptors**	
No	11 (35.5)
Yes	20 (64.5)
**PET-FDG ^3^**	
No	20 (76.9)
Yes	6 (23.1)
Unknown/missing	5
**First line therapy**	
None	8 (25.8)
Somatostatin	14 (45.2)
Chemotherapy	5 (16.2)
Biological therapy and somatostatin	1 (3.2)
Best supportive care	1 (3.2)
Local therapy	2 (6.4)
**Progression after first line therapy**	
No	11 (35.5)
Yes	20 (58.1)
**Grading**	
G1	15 (48.4)
G2	14 (45.2)
G3	2 (6.4)

^1^: iNET: ileal neuroendocrine tumours; ^2^: SSA Receptors: somatostatin analogs receptors; ^3^: PET-FDG: fluorodeoxyglucose (FDG)-positron emission tomography (PET).

**Table 2:** Demographic and clinical features of pNET ^1^ patients.

	N. (%)
**Age**, years: median value (range, IQR)	63 (25–79, 49–69)
<70	46 (78.0)
≥70	13 (22.0)
**Gender**	
Female	29 (49.1)
Male	30 (50.9)
**TNM at diagnosis**	
I	6 (10.2)
II	17 (28.8)
III	17 (28.8)
IV	19 (32.2)
**Metastases at diagnosis**	
No	40 (67.8)
Yes	19 (32.2)
**Radical surgery**	
No	17 (28.8)
Yes	42 (71.2)
**Metastatic sites**	
None	40 (67.8)
Liver	15 (25.4)
Lymphnodes	2 (3.4)
Peritoneum	1 (1.7)
Other	1 (1.7)
**Ki67 (%)**	
<3	31 (52.5)
3–20	21 (35.6)
>20	7 (11.9)
**Relapse**	
No	31 (52.5)
Yes	11 (18.6)
Liver	7 (63.6)
Lymphnodes	2 (18.2)
Other	2 (18.2)
Unknown/missing	17 (28.8)
**SSA Receptors** ^2^	
No	1 (1.7)
Yes	21 (35.6)
Unknown/missing	37 (62.7)
**PET-FDG** ^3^	
No	8 (13.6)
Yes	12 (20.3)
Unknown/missing	39 (66.1)
**Grading**	
G1	36 (61.0)
G2	17 (28.8)
G3	6 (10.2)
**First line therapy**	
None	29 (49.1)
Chemotherapy	10 (17.0)
Everolimus	2 (3.4)
Radiometabolic therapy	3 (5.1)
Somatostatin analogues	15 (25.4)
**Progression after first line therapy**	
No	39 (66.1)
Yes	20 (33.9)

^1^: pNET: pancreatic neuroendocrine tumours; ^2^: SSA Receptors: somatostatin analogs receptors; ^3^: PET-FDG: fluorodeoxyglucose (FDG)-positron emission tomography (PET).

**Table 3:** Univariate analysis of Overall Survival in iNET. Statistically significant values are reported in bold. nr: not reached.

	N. Patients	N. Deaths	Median OS ^1^ (Months) (95% CI)	HR ^2^ (95% CI ^3^)	*p*
**Overall**	31	12	138 (72-nr)	-	-
**Age** (continuous variable)	31	12	-	1.051 (0.99–1.107)	0.055
<70	22	8	138 (75-nr)	1.00	
≥70	9	4	nr	2.24 (0.63–8.00)	0.215
**Gender**					
Female	16	5	nr	1.00	
Male	15	7	138 (17-nr)	1.46 (0.44–4.83)	0.530
**Stage at diagnosis**					
II	5	2	138 (38-nr)	1.00	
III	11	3	nr	0.96 (0.16–5.81)	
IV	15	7	nr	1.98 (0.39–10.14)	0.505
**Metastases at diagnosis**					
No	16	5	nr	1.00	
Yes	15	7	nr	2.03 (0.62–6.69)	0.242
**Radical Surgery**					
No	13	7	111 (18-nr)	1.00	
Yes	18	5	nr	0.35 (0.10–1.22)	0.100
**Ki67 (%)**					
<3	15	4	nr	1.00	
3–20	14	6	111 (18-nr)	2.16 (0.59–7.86)	
>20	2	2	21 (8-nr)	11.58 (1.79–74.73)	**0.036**
**SSA Receptors ^4^**					
No	11	5	nr	1.00	
Yes	20	7	138 (75-nr)	0.65 (0.20–2.10)	0.472
**PET-FDG ^5^**					
No	20	8	138 (34-nr)	1.00	
Yes	6	1	nr	0.64 (0.08–5.36)	0.682
**% hERG1 positive cells**					
Negative (<40)	7	5	72 (6-nr)	1.00	
Positive (≥40)	24	7	nr	0.23 (0.07–0.80)	**0.020**

^1^: OS: Overall Survival; ^2^: HR: Hazard Ratio; ^3^: CI: Confidence Interval; ^4^: SSA Receptors: somatostatin analogs receptors; ^5^: PET-FDG: fluorodeoxyglucose (FDG)-positron emission tomography (PET).

**Table 4:** Percentage of hERG1 positive tumour cells in relation to baseline characteristics in pNET patients. Statistically significant values are reported in bold.

	% hERG1 Positive Tumour Cells	
	**Median Value (Range)**	***p***
**Overall**	13 (0–90)	**-**
**Age**		
<70	14 (0–90)	
≥70	8 (0–80)	0.808
**Gender**		
Female	25 (0–80)	
Male	9 (0–90)	0.053
**TNM at diagnosis**		
I	7 (0–80)	
II	40 (0–90)	
III	0 (0–80)	
IV	8 (0–90)	**0.011**
**Metastases at diagnosis**		
No	15 (0–90)	
Yes	8 (0–90)	0.196
**Radical Surgery**		
No	8 (0–90)	
Yes	15 (0–90)	0.439
**Ki67 (%)**		
<3	20 (0–90)	
3–20	12 (0–80)	
>20	0 (0–90)	0.292
**SSA Receptors ^1^**		
No	0 (-)	
Yes	12 (0–60)	0.317
**PET-FDG ^2^**		
No	5 (0–15)	
Yes	0 (0–80)	0.374
**Grading**		
G1	20 (0–90)	
G2	12 (0–80)	
G3	0 (0–90)	0.128

^1^: SSA Receptors: somatostatin analog receptors; ^2^: PET-FDG: fluorodeoxyglucose (FDG)-positron emission tomography (PET).

**Table 5:** Univariate analysis of Overall Survival in pancreatic NET patients.

	N. pts	N. Deaths	Median OS ^1^ (Months) (95% CI ^2^)	P (Logrank)	HR ^3^ (95% CI)	P (Cox)
**Overall**	59	10	nr	-	-	-
**Age** (continuous variable)	59	10	-	-	0.977 (0.937–1.019)	0.271
<70	46	10	nr		1.00	
≥70	13	0	nr	0.076	Ne	-
**Gender**						
Female	29	2	nr		1.00	
Male	30	8	nr	0.040	4.43 (0.94–20.93)	0.060
**Stage at diagnosis**						
I	6	0	nr		Ne	
II	17	0	nr		1.00	
III	17	2	nr		Ne	
IV	19	8	112 (14-nr)	0.001	Ne	-
**Metastases at diagnosis**						
No	40	2	nr		1.00	
Yes	19	8	112 (14-nr)	0.0001	11.27 (2.38–53.32)	0.002
**Radical Surgery**						
No	17	7	nr		1.00	
Yes	42	3	nr	0.0003	0.12 (0.03–0.48)	0.002
**Ki67 (%)**						
<3	31	2	nr		1.00	
3–20	21	3	nr		2.63 (0.44–15.82)	
>20	7	5	7 (3-nr)	<0.0001	51.17 (8.64–303.09)	<0.0001
**SSA Receptors ^4^**						
No	1	1	3 (-)		1.00	
Yes	21	5	nr	<0.0001	Ne	-
**PET-FDG ^5^**						
No	8	3	112 (14-nr)		1.00	
Yes	12	4	nr	0.975	1.02 (0.23–4.58)	0.975
**% hERG1 positive cells**						
Negative (<40)	41	9	nr		1.00	
Positive (≥40)	18	1	nr	0.134	0.23 (0.03–1.85)	0.169

^1^: OS: Overall Survival; ^2^: HR: Hazard Ratio; ^3^: CI: Confidence Interval; ^4^: SSA Receptors: somatostatin analogs receptors; ^5^: PET-FDG: fluorodeoxyglucose (FDG)-positron emission tomography (PET).

**Table 6:** Univariate analysis of Overall Survival in the whole NET cohort (iNETs + pNETs). Statistically significant values are reported in bold. nr: not reached.

	N. Patients	N. Deaths	Median OS ^1^ (Months) (95% CI ^2^)	HR (95% CI)	*p*
**OVERALL**					
**% hERG1 positive cells**					
Negative (<40)	48	14	nr	1.00	
Positive (≥40)	42	8	nr	0.60 (0.25–1.43)	0.248
**TNM IV**					
**% hERG1 positive cells**					
Negative (<40)	21	13	72 (10-nr)	1.00	
Positive (≥40)	13	2	nr	0.22 (0.05–0.97)	**0.046**
**TNM I-III**					
**% hERG1 positive cells**					
Negative (<40)	27	1	96 (88–100)	1.00	
Positive (≥40)	29	6	86 (73–99)	5.18 (0.62–43.36)	0.129

^1^: OS: Overall Survival; ^2^: CI: Confidence Interval.

## Data Availability

Data are available upon request.

## Supplementary Materials

The following supporting information can be downloaded at: https://www.mdpi.com/article/10.3390/ijms231810623/s1.
