# Supplementary material for: Prognostic role of hERG1 Potassium Channels in Neuroendocrine Tumours of the Ileum and Pancreas
Source: Int J Mol Sci. 2022 Sep 13;23(18):10623. doi: 10.3390/ijms231810623 (PMC9504580; doi:10.3390/ijms231810623)
Supplement: Supplementary file 1 [file ijms-23-10623-s001.zip › ijms-1870714-supplementary.pdf]

Supplementary Materials

Prognostic role of hERG1 potassium channels in neuroendocrine tumours of the ileum and pancreas.

Jessica Iorio[1@], Lorenzo Antonuzzo[1,2@], Emanuela Scarpi[3], Massimo D'Amico[4], Claudia Duranti[1], Luca Messerini[1], Clotilde Sparano[5], Damiano Caputo[6,7], Daniele Lavacchi[1,2], Domenico Borzomati[6,7], Alice Antonelli[1,2], Lorenzo Nibid[7,8], Giuseppe Perrone[7,8], Alessandro Coppola[6,7], Roberto Coppola[6,7], Francesco di Costanzo[2], Elena Lastraioli[1,9*], Annarosa Arcangeli[1,9]

[1]Department of Experimental and Clinical Medicine, University of Florence, Florence Italy
[2]Medical Oncology, Azienda Ospedaliero-Universitaria Careggi, Florence, Italy
[3]Unit of Biostatistics and Clinical Trials, IRCCS Istituto Romagnolo per lo Studio dei tumori (IRST) "Dino Amadori", Meldola (FC), Italy
[4]DI.V.A.L Toscana Srl, Sesto Fiorentino (Florence), Italy

[5]Endocrinology Unit, Department of Experimental and Clinical Biomedical Sciences "Mario Serio", University of Florence, Florence Italy

[6]General Surgery, Campus Bio-Medico University, Rome, Italy
[7] Fondazione Policlinico Universitario Campus Bio-Medico, Rome, Italy

[8]Pathology Unit, Campus Bio-Medico University, Rome, Italy
[9]Complex Dynamics Study Centre (CSDC), University of Florence, Florence Italy

[@]Equally contributed
*Correspondence: elena.lastraioli@unifi.it; +39 0552751319

Figure S1. *Kaplan-Meier plots of OS in ileal NET patients according to gender and age.*

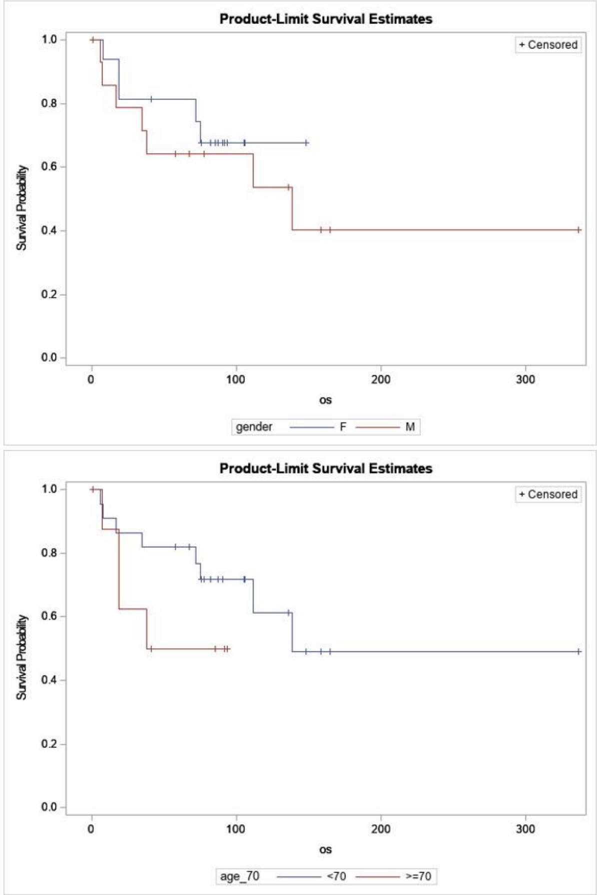

The SAS System

Obs	age_70	gender	os	dec
1	<70	F	7.86	1
2	<70	F	71.97	1
3	<70	F	75.10	1
4	<70	F	75.53	0
5	<70	F	81.97	0
6	<70	F	87.11	0
7	<70	F	90.26	0
8	<70	F	105.16	0
9	<70	F	105.46	0
10	<70	F	148.03	0
11	<70	M	5.92	1
12	<70	M	16.94	1
13	<70	M	34.44	1
14	<70	M	57.96	0
15	<70	M	67.04	0
16	<70	M	77.80	0
17	<70	M	111.25	1
18	<70	M	135.72	0
19	<70	M	138.36	1
20	<70	M	157.99	0
21	<70	M	164.47	0
22	<70	M	336.35	0
23	>=70	F	18.36	1
24	>=70	F	18.49	1
25	>=70	F	41.15	0
26	>=70	F	85.16	0
27	>=70	F	91.68	0
28	>=70	F	93.55	0
29	>=70	M	0.63	0
30	>=70	M	6.91	1
31	>=70	M	37.86	1

Table S1. *Percentage of hERG1 positive tumour cells in relation to baseline characteristics in ileal NET patients.*

	**% hERG1 positive tumor cells**	
	**Median value (range)**	**p**
**Overall**	80 (0-100)	
**Age**		
<70	80 (0-100)	
≥70	90 (0-100)	0.420
**Gender**		
Female	80 (0-100)	
Male	90 (0-100)	0.496
**TNM at diagnosis**		
II	45 (0-100)	
III	90 (0-100)	
IV	80 (0-100)	0.289
**Metastases at diagnosis**		
No	90 (0-100)	
Yes	80 (0-100)	0.233
**Radical Surgery**		
No	80 (0-100)	
Yes	85 (0-100)	0.549
**Ki67 (%)**		
<3	80 (0-100)	
3-20	90 (0-100)	
>20	0 (0-0)	0.210
**SSA Receptors**[1]		
No	90 (0-100)	
Yes	80 (0-100)	0.509
**PET-FDG**[2]		
No	80 (0-100)	
Yes	90 (0-100)	0.306
**Grading**		
G1	80 (0-100)	
G2	90 (0-100)	
G3	0 (0-0)	0.210

[1] *SSA Receptors: somatostatin analogs receptors.*

[2] *PET-FDG: fluorodeoxyglucose (FDG)-positron emission tomography (PET).*

***Table S2**. Univariate analysis of Overall Survival in TNM stage IV iNET. Statistically significant values are reported in bold. nr: not reached*

	N. patients	N. deaths	Median OS[3] (months) (95% CI[4])	HR[5] (95% CI)	P
**Overall**	15	7	nr	-	-
**Age** (continuous variable)	15	7	-	1.017 (0.957-1.080)	0.592
<70	12	6	nr	1.00	
≥70	3	1	nr	0.72 (0.08-6.05)	0.761
**Gender**					
Female	11	4	nr	1.00	
Male	4	3	26 (6-nr)	3.40 (0.74-15.56)	0.114
**Metastases at diagnosis**					
No	0	-	-	1.00	
Yes	15	7	nr	-	-
**Radical Surgery**					
No	12	6	75 (8-nr)	1.00	
Yes	3	1	nr	0.63 (0.07-5.23)	0.667
**Ki67 (%)**					
<3	4	1	nr	1.00	
3-20	9	4	nr	2.40 (0.27-21.58)	
>20	2	2	21 (8-nr)	11.08 (0.87-141.59)	0.137
**SSA Receptors**[6]					
No	3	2	18 (8-nr)	1.00	
Yes	12	5	nr	0.44 (0.08-2.31)	0.332
**PET-FDG**[7]					
No	10	5	nr	1.00	
Yes	3	1	nr	0.85 (0.10-7.30)	0.881
**% hERG1 positive cells**					
Negative (<40)	5	5	34 (6-nr)	1.00	
Positive (≥40)	10	2	nr	0.12 (0.02-0.65)	**0.013**

[3] *OS: Overall Survival.*

[4] *HR: Hazard Ratio.*

[5] *CI: Confidence Interval*

[6] *SSA Receptors: somatostatin analogs receptors.*

[7] *PET-FDG: fluorodeoxyglucose (FDG)-positron emission tomography (PET).*

Table S3. *Univariate analysis of Progression Free Survival in TNM I-III ileal NET patients. Statistically significant values are reported in bold.*

	N. pts	N. events	% 5-years PFS[8] (95% CI[9])	P (logrank)	HR[10] (95% CI)	P (Cox)
**Overall**	16	10	30 (6-54)		-	-
**Age** (continuous variable)	16	10	-	-	0.975 (0.928-1.024)	0.312
<70	10	7	30 (2-58)		1.00	
≥70	6	3	27 (0-71)	0.605	0.70 (0.18-2.72)	0.607
**Gender**						
Female	5	2	53 (5-100)		1.00	
Male	11	8	20 (0-45)	0.198	2.67 (0.56-12.66)	0.215
**Metastases at diagnosis**						
No	0	-	-		1.00	
Yes	15	9	30 (6-54)	-	-	-
**Radical Surgery**						
No	1	1	0		1.00	
Yes	15	9	32 (7-58)	0.308	0.34 (0.04-3.02)	0.331
**Ki67 (%)**						
<3	11	6	35 (4-66)		1.00	
3-20	5	4	20 (0-55)		1.65 (0.46-5.94)	
>20	0	-	-	0.438	-	0.442
**SSA Receptors**[11]						
No	8	5	29 (0-62)		1.00	
Yes	8	5	33 (0-69)	0.870	1.11 (0.32-3.86)	0.870
**PET-FDG**[12]						
No	10	8	13 (0-37)		1.00	
Yes	3	0	100	**0.029**	ne	-
**% hERG1 positive cells**						
Negative (<40)	2	0	100		1.00	
Positive (≥40)	14	10	23 (0-46)	0.164	ne	-

[8] *PFS: Progression Free Survival.*

[9] *HR: Hazard Ratio.*

[10] *CI: Confidence Interval*

[11] *SSA Receptors: somatostatin analogs receptors.*

[12] *PET-FDG: fluorodeoxyglucose (FDG)-positron emission tomography (PET).*

Table S4. *Univariate analysis of Progression Free Survival in TNM IV ileal NET patients.*

	N. pts	N. events	Median PFS[13] (months) (95% CI[14])	P (logrank)	HR[15] (95% CI)	P (Cox)
**Overall**	15	14	6 (3-9)	-	-	-
**Age** (continuous variable)	15	14	-	-	0.977 (0.933-1.023)	0.327
<70	12	12	6 (3-13)		1.00	
≥70	3	2	6 (2-nr)	0.441	0.55 (0.12-2.54)	0.446
**Gender**						
Female	11	10	7 (3-16)		1.00	
Male	4	4	3 (0-nr)	0.187	2.22 (0.66-7.53)	0.199
**Metastases at diagnosis**						
No	0	-	-		1.00	
Yes	15	14	6 (3-9)	-	ne	-
**Radical Surgery**						
No	12	11	6 (2-16)		1.00	
Yes	3	3	5 (3-nr)	0.332	1.94 (0.50-7.61)	0.340
**Ki67 (%)**						
<3	4	3	12 (5-nr)2		1.00	
3-20	9	9	3 (0-9)		2.66 (0.70-10.05)	
>20	2	2	9 (5-nr)	0.323	1.81 (0.29-11.26)	0.347
**SSA Receptors**[16]						
No	3	3	5 (2-nr)		1.00	
Yes	12	11	7 (3-16)	0.077	0.28 (0.06-1.26)	0.098
**PET-FDG**[17]						
No	10	9	6 (0-13)		1.00	
Yes	3	3	3 (3-nr)	0.217	2.42 (0.57-10.30)	0.231
**% hERG1 positive cells**						
Negative (<40)	5	5	9 (3-nr)		1.00	
Positive (≥40)	10	9	5 (0-8)	0.765	1.19 (0.38-3.68)	0.765

[13] *PFS: Progression Free Survival.*

[14] *HR: Hazard Ratio.*

[15] *CI: Confidence Interval*

[16] *SSA Receptors: somatostatin analogs receptors.*

[17] *PET-FDG: fluorodeoxyglucose (FDG)-positron emission tomography (PET).*

Figure S2. *Kaplan-Meier plots of PFS in ileal NET patients according to hERG1 expression (cut-off: 40%). Blue curves: hERG1 negative samples (<40%), Red curves: hERG1 positive samples (≥40%).*

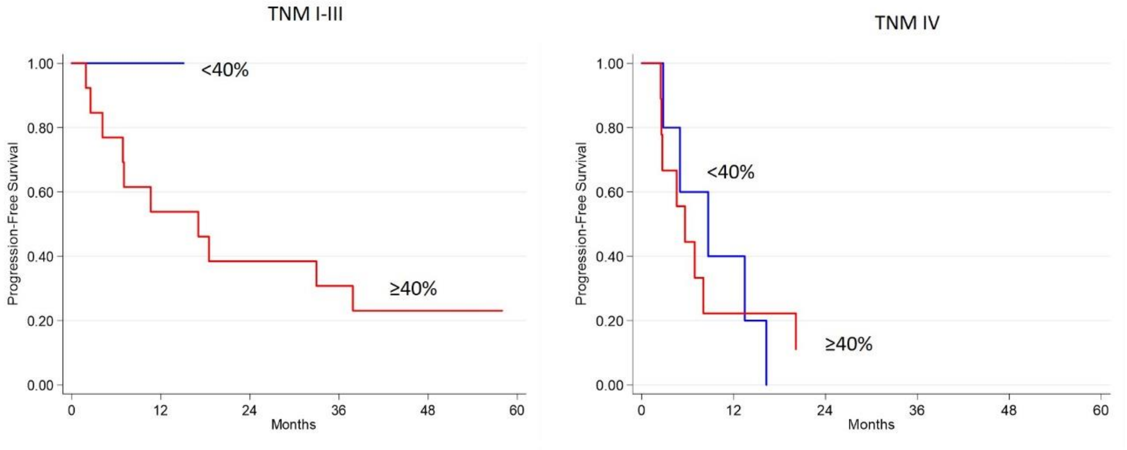

Table S5. *Univariate analysis of Progression Free Survival in TNM I-III pancreatic NET patients. Statistically significant values are reported in bold.*

	N. pts	N. events	% 5-years PFS[18] (95% CI[19])	P (logrank)	HR[20] (95% CI)	P (Cox)
**Overall**	40	9	76 (63-90)	-	-	-
**Age** (continuous variable)						
<70	30	8	71 (55-88)		1.00	
≥70	10	1	90 (71-100)	0.240	0.31 (0.04-2.46)	0.267
**Gender**						
Female	22	4	82 (65-98)		1.00	
Male	18	5	69 (47-92)	0.500	1.57 (0.42-5.84)	0.503
**Metastases at diagnosis**						
No	40	9	76 (63-90)		1.00	
Yes	0	-	-	-	ne	-
**Radical Surgery**						
No	0	-	-		1.00	
Yes	40	9	76 (63-90)	-	ne	-
**Ki67 (%)**						
<3	26	3	88 (76-100)		1.00	
3-20	13	5	55 (26-85)		ne	
>20	1	1	0	**<0.0001**	ne	-
**SSA Receptors**[21]						
No	0	-	-		1.00	
Yes	10	6	27 (0-59)	-	ne	-
**PET-FDG**[22]						
No	3	1	67 (13-100)		1.00	
Yes	4	2	50 (1-99)	0.829	1.31 (0.11-15.21)	0.829
**% hERG1 positive cells**						
Negative (<40)	25	6	73 (55-92)		1.00	
Positive (≥40)	15	3	80 (60-100)	0.734	0.79 (0.20-3.15)	0.734

[18] *PFS: Progression Free Survival.*

[19] *HR: Hazard Ratio.*

[20] *CI: Confidence Interval*

[21] *SSA Receptors: somatostatin analogs receptors.*

[22] *PET-FDG: fluorodeoxyglucose (FDG)-positron emission tomography (PET).*

Table S6. *Univariate analysis of Progression Free Survival in TNM IV pancreatic NET patients. Statistically significant values are reported in bold.*

	N. pts	N. events	Median PFS[23] (months) (95% CI[24])	P (logrank)	HR[25] (95% CI)	P (Cox)
**Overall**	19	16	9 (4-31)	-	-	-
**Age** (continuous variable)						
<70	16	14	8 (4-31)		1.00	
≥70	3	2	10 (2-nr)	0.821	0.84 (0.19-3.77)	0.822
**Gender**						
Female	7	5	74 (2-nr)		1.00	
Male	12	11	8 (2-22)	0.150	2.32 (0.71-7.57)	0.162
**Metastases at diagnosis**						
No	0	-	-		1.00	
Yes	19	16	9 (4-31)	-	ne	-
**Radical Surgery**						
No	17	14	8 (2-74)		1.00	
Yes	2	2	16 (10-nr)	0.855	1.15 (0.25-5.30)	0.856
**Ki67 (%)**						
<3	5	5	22 (5-nr)		1.00	
3-20	8	5	20 (2-nr)		0.95 (0.25-3.56)	
>20	6	6	4 (1-nr)	**0.018**	4.72 (1.13-19.66)	**0.038**
**SSA Receptors**[26]						
No	1	1	2 (-)		1.00	
Yes	11	9	22 (5-74)	**0.0009**	ne	-
**PET-FDG**[27]						
No	5	3	31 (8-nr)		1.00	
Yes	8	8	5 (2-10)	0.043	3.77 (0.95-14.90)	0.058
**% hERG1 positive cells**						
Negative (<40)	16	14	10 (5-31)		1.00	
Positive (≥40)	3	2	4 (1-nr)	0.910	1.09 (0.24-4.90)	0.909

[23] *PFS: Progression Free Survival.*

[24] *HR: Hazard Ratio.*

[25] *CI: Confidence Interval*

[26] *SSA Receptors: somatostatin analogs receptors*

[27] *PET-FDG: fluorodeoxyglucose (FDG)-positron emission tomography (PET).*

Figure S3. *Kaplan-Meier plots of PFS in pancreatic NET patients according to hERG1 expression (cut-off: 40%) in TNM I-III (left) and TNM IV patients (right). Blue curves: hERG1 negative samples (<40%), Red curves: hERG1 positive samples (≥40%).*

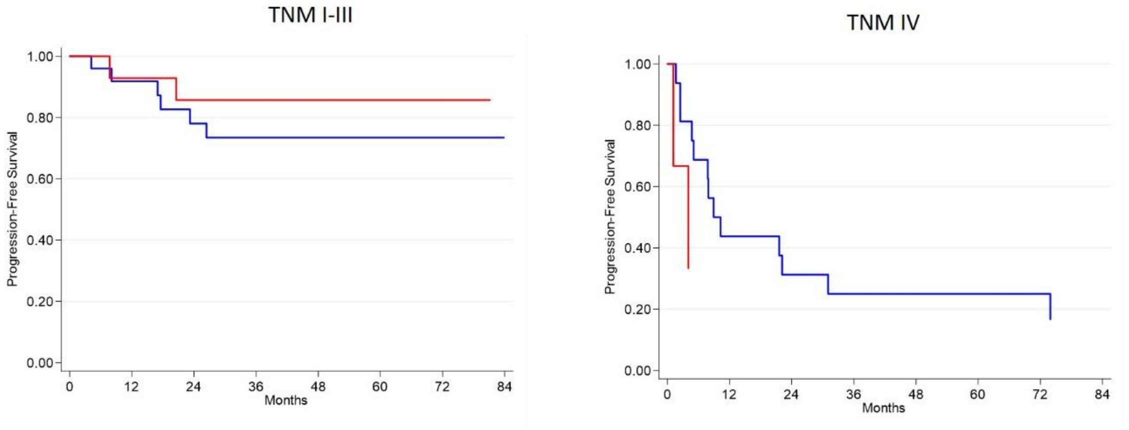

Table S7. *P values of the comparison between control and E4031- treated INS 1E.*

		P value
CTR vs 0.1 µM	24h	0.002
	48h	0.040
	72h	0.020
	96h	0.004
CTR vs 1 µM	24h	0.013
	48h	0.030
	72h	< 0.001
	96h	< 0.001
CTR vs 10 µM	24h	0.004
	48h	0.010
	72h	< 0.001
	96h	< 0.001
CTR vs 25 µM	24h	0.002
	48h	0.011
	72h	0.001
	96h	< 0.001
CTR vs 50 µM	24h	< 0.001
	48h	0.007
	72h	< 0.001
	96h	< 0.001
CTR vs 100 µM	24h	0.001
	48h	0.006
	72h	< 0.001
	96h	< 0.001
CTR vs 200 µM	24h	0.002
	48h	0.001
	72h	< 0.001
	96h	< 0.001
